# JLVEA: Lightweight Real-Time Video Stream Encryption Algorithm for Internet of Things

**DOI:** 10.3390/s20133627

**Published:** 2020-06-28

**Authors:** Junhyeok Yun, Mihui Kim

**Affiliations:** School of Computer Engineering & Applied Mathematics, Computer System Institute, Hankyong National University, Jungang-ro, Anseong-si, Gyeonggi-do 17579, Korea; junhyeok2723@hknu.ac.kr

**Keywords:** security, privacy, IoT, video encryption, lightweight encryption

## Abstract

Along with the recent growth of Internet of Things (IoT) security camera market, there have been a number of personal information leakage incidents from security attacks targeting such cameras. Therefore, a permutation-based video encryption algorithm was proposed to secure video streams in low-performance processors such as IoT security cameras. However, existing permutation-based video encryption algorithms are vulnerable to known-plaintext attacks since they use the same permutation list for every frame. Moreover, situation deduction based on the color composition is possible. In this paper, we propose a new permutation-based video encryption algorithm that updates the permutation list for every frame using a crypto secure pseudo-random number generator without significantly increasing memory usage. By doing so, the algorithm becomes robust to known-plaintext attacks, which has been a common problem with existing permutation-based video encryption algorithms. In addition, color channel separation can prevent attackers from deducing situations through color composition. Pre-compression encryption is applied to make the algorithm robust to data loss because of packet loss. We implement the proposed algorithm and conduct an experiment to show its performance in terms of probability of data loss because of packet loss, encryption speed, and memory usage.

## 1. Introduction

Internet of Things (IoT) security camera is a type of IoT sensor device that captures video from a place in need of surveillance and streams it to outside users in real time [1]. Since an IoT security camera always captures video, the risk of personal information leakage is high [2]. Recently, the number of personal information leakage incidents from security attacks to IoT security cameras has increased. Thus, IoT security cameras should include a security mechanism to block access from unauthorized entities. Access control mechanisms [3,4] and video stream encryption algorithms [5,6] have been proposed to keep the video stream safe from an attack. Access control mechanisms can block an attack with a small processing overhead [7]. However, it requires continuous management by users, such as a changing password or access control list management. Additionally, it is vulnerable to sniffing attacks [8]. Therefore, a video stream protection mechanism through video stream encryption has been proposed [9]. The video stream encryption algorithms for an IoT security camera should satisfy its real-time requirements.

IoT security cameras use a low-power processor because they should always be activated [10]. The low-power processor generally has a lower performance than standard computer processors [11]. Existing block cipher algorithms (e.g., advanced encryption standard (AES) [12] and SEED [13]) are designed for text data encryption. However, video stream data is generally larger in size than text data. Video stream encryption using block cipher algorithms cause high processing overhead. Thus, block-structured encryption algorithms are not appropriate for encrypting video streams on an IoT security camera that includes a low-power processor. High processing overhead causes delays in the video stream, and repetitive delays prevent a user from accessing the video stream in real-time.

To solve the massive processing overhead problem of video stream encryption using a block cipher algorithm, Liu and Koenig [14] proposed a permutation-based video encryption algorithm. This algorithm encrypts a video frame by permutating one specific part with another specific part in the frame. This algorithm can encrypt a video stream with 93.75% lesser processing overhead than AES. Thus, the permutation-based video encryption algorithm is appropriate for encrypting the video stream from IoT security cameras. However, this algorithm is vulnerable to known-plaintext attacks since it uses the same permutation list for every frame of the video stream, and attackers can partly recover original frame data using file structures such as a header marker or end-of-file (EOF) marker, as the algorithm encrypts the video stream after compression. Moreover, if the critical file structure such as a header marker or EOF marker is lost because of packet loss while real-time streaming, the user cannot receive the complete frame data. Sultana and Shubhangi [15] proposed a permutation-based video encryption algorithm based on the faro shuffle algorithm. This algorithm is robust to a file structure inference attack since it encrypts the video stream before compression. However, it is also vulnerable to known-plaintext attacks since it uses the same permutation list for every frame. Moreover, it is vulnerable to brute-force attacks since the complexity of the faro shuffle algorithm is low.

In this paper, we propose an improved permutation-based video encryption algorithm that has low processing overhead and high security. To avoid a known-plaintext attack, which is a common problem with permutation-based video encryption algorithms, the proposed algorithm updates the permutation list for each frame using crypto secure pseudo-random number generator (CSPRNG). In addition, the proposed algorithm improves security through separate color channels.

## 2. Related Work

### 2.1. Block Cipher-Based Video Encryption

**Figure 1:**
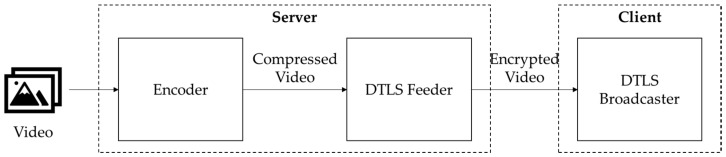
Datagram transport layer security (DTLS) encryption flow.

Block cipher-based video encryption algorithms that work on lightweight protocols (i.e., user datagram protocol (UDP) and constrained application protocol (CoAP) [16]) have been suggested for real-time video stream encryption on low-performance devices. The datagram transport layer security (DTLS) algorithm is an UDP-based lightweight video stream encryption algorithm (see Figure 1) [17]. On server, the encoder compresses the video stream with the H.264-based real-time messaging protocol (RTMP) [18] and sends it to the DTLS feeder. The DTLS feeder then encrypts the video stream with an AES256 encryption algorithm and transfers it to the client. On client, a DTLS broadcaster decrypts the received video stream. However, DTLS also has a high processing overhead on low-performance processors because it uses the AES256 block cipher algorithm. Thus, DTLS is not appropriate for real-time video stream encryption on IoT security cameras.

To address the processing overhead problem, a hardware-based AES encryption mechanism has been proposed in [19]. Here, an AES-specialized hardware chipset conducts the encryption in various steps. Hardware-based AES encryption can address processing overhead problems on a lightweight processor. However, it has low versatility and requires additional cost. To solve the problems of block cipher-based video stream encryption algorithms, a permutation-based video encryption algorithm, which encrypts video stream only using the information inside the frame, is proposed.

### 2.2. Permutation-based Video Encryption

A permutation-based video encryption algorithm encrypts video by permutating a specific part in the frame with another specific part. Video frame data includes a significant amount of pixel information. Thus, recovering the original location of all pixels is almost impossible. For full high definition (FHD) resolution, which is mostly adopted for multimedia contents, a frame includes 1920×1080=2,073,600 pixels. In this instance, a malicious attacker must rearrange and review 2,073,600 frames to find the original frame using a brute-force attack. Thus, brute-force attack on a permutated frame without the permutation list is practically impossible.

The encryption speed of permutation-based video encryption is much faster than the block cipher-based encryption. Generally, the size of video data is significantly larger than text data. Block cipher-based encryption brings high overhead since it performs an operation at the same part of data repeatedly. Thus, adopting block cipher-based video encryption on real-time video streaming is practically impossible. Permutation-based video encryption algorithms do not perform permutation at the same part of data repeatedly. Thus, it can encrypt video with lower overhead than block cipher-based video encryption.

**Figure 2:**
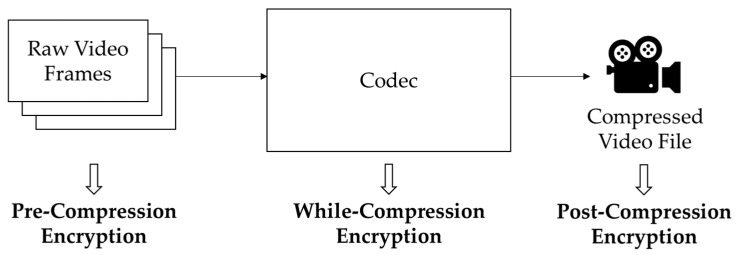
Classification of permutation-based video encryption algorithms.

The position of the encryption algorithm classifies the permutation-based video encryption algorithms (see Figure 2). A video codec compresses the original video stream. Permutation-based video encryption algorithms can be classified as pre-compression, while-compression, and post-compression.

Liu and Koenig [14], Sultana and Shubhangi [15], and Akhter et al. [20] are previously proposed permutation-based video encryption algorithms (see Table 1). The while-compression encryption algorithm by Akhter et al. performs encryption simultaneously with the moving picture experts group (MPEG) video compression [20]. This algorithm encrypts the video before entropy coding and after discrete cosine transform (DCT) conversion. By doing so, it can reduce encryption time and minimize the size of final cryptographic videos. When the DCT conversion is complete, the high-frequency components within the video frame are removed. Thus, this algorithm is advantageous in terms of encryption speed and the size of the cryptographic video is smaller than the original video. However, it has to use a special codec that includes encryption algorithms, and is not compatible with other video formats except MPEG. Moreover, it is vulnerable to known-plaintext attacks using the same permutation list for all frames in the video.

The algorithm proposed by Liu and Koenig is a post-compression encryption algorithm that separates a compressed video frame into blocks of a specific size, and then permutates them to encrypt videos [14]. Since the compressed video has a smaller size than the original video, the size of the final cryptographic video can also be reduced. However, the attacker can restore the original frame data using file structures such as header markers, an EOF marker, and Huffman tables. Moreover, if some permutation list is missing during the decryption, the critical file structure may be lost. In this case, the client cannot even obtain a portion of the original frame data that has not been lost. This algorithm divides the compressed frame data into 256 same-sized blocks and permutates them according to the permutation list for encrypting the video frames. However, it is vulnerable to known-plaintext attacks as it uses the same permutation list for all frames in the video. In order to counter known-plaintext attacks, a method for creating and using different permutation lists for each frame was proposed. However, it is unsuitable for real-time streaming because of the high processing overhead of key exchange and permutation list generation.

The algorithm proposed by Sultana and Shubhangi [15] is a pre-compression encryption algorithm. Although pre-compression encryptions are less effective and result in large sized final cryptographic frames by including high-frequency components in the original frame, it is impossible to deduce the original video frame data based on the file structure such as the header marker and EOF marker. Moreover, even if some data goes missing during the transmission, the content of the video frame can still be verified except for the missing parts. Since this algorithm is designed to repeat the faro shuffle and frame rotation several times instead of generating a random permutation list, it is relatively easy to restore the original frame data through brute-force attacks.

In this paper, while maintaining the efficiency of existing permutation-based algorithms, we propose a robust permutation-based video encryption algorithm for known-plaintext attacks, which is a common vulnerability of such algorithms. CSPRNG is used to update the permutation lists for each video frame. Instead of generating a permutation list for each frame, the random values generated by CSPRNG are added to the permutation list used in the previous frame. Accordingly, we can solve the problem of key exchanging and permutation list generating overhead, a weakness in Liu and Koenig’s algorithm [14]. In addition, by performing permutation for each color channel following color channel separation, non-recognition of shape elements and color elements can be simultaneously satisfied. Making color elements unrecognizable can lead to a higher security than the previously proposed permutation-based video encryption algorithms, which only make the shape elements unrecognizable.

## 3. Proposed Algorithm

The proposed algorithm was based on the permutation-based video encryption mechanism; it included a permutation list management module to resolve the vulnerabilities of the previously proposed permutation-based video encryption algorithms, and a video processing module to improve security. The permutation list management module stores and updates the permutation list for each frame using CSPRNG. The image processing module performs the permutation on the video frame, including color channel permutation and pixel permutation, using the permutation list. The communication module performs the seed exchange and the cryptographic video frame exchange.

### 3.1. Encryption Module Structure

**Figure 3:**
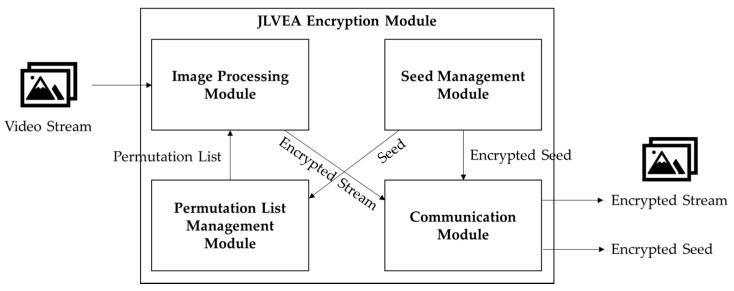
JLVEA encryption module structure and flows.

**Figure 4:**
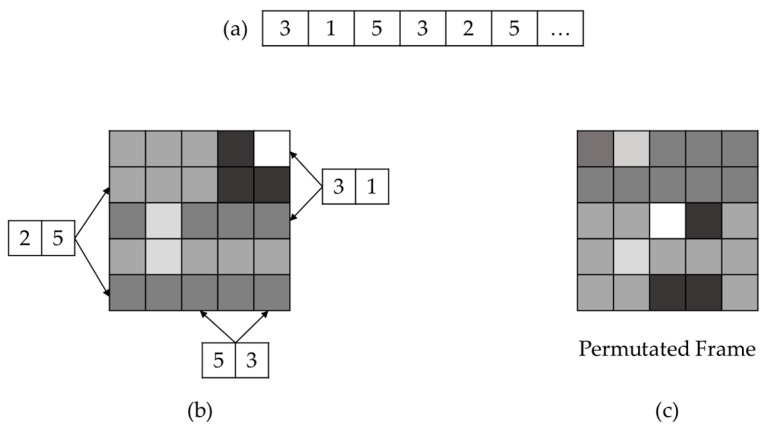
Matrix permutation in video frame: (**a**) permutation list—image processing module permutates the video frame based on permutation list; (**b**) matrix permutation—image processing module permutates rows and columns using every two elements from permutation list; and (**c**) permutated frame.

The jumble lightweight video encryption algorithm (JLVEA) encryption module includes a seed management module, a permutation list management module, an image processing module, and a communication module (see Figure 3). The seed management module generates and stores a seed for permutation lists generation. The seed is a 128-bit integer value, which takes about 5849 years in a computing environment with 1 tera floating point operations per second (TFLOPS) to deduce by a brute-force attack. The seed value directly affects the permutation lists generation. Thus, it must be kept safe after the exchange. All access attempts by external entities without the permutation list management module is restricted. For a more robust seed protection, seed management modules can be located in physically isolated environments, such as trusted platform modules (TPMs) [21]. The permutation list management module generates the permutation list using CSPRNG and the seed. The generated permutation list is stored within the permutation list management module. The resolution of the input video determines the length of the permutation list. The image processing module loads the original video data and performs matrix permutation on each frame of the video based on the permutation list (see Figure 4). At this time, the color channel of the input video frame is separated and reordered so that the color composition of the original video frame cannot be deduced. The separation of color channels makes it difficult for humans to visually identify the contents of the original video frame with a relatively small number of permutations. The video stream server and client determine the color channel order based on the exchanged seed values (see Table 2). After exchanging the seed, they calculate “SEED mod 5” to get a color channel reordering information. Alternatively, they can use the seed values and CSPRNG to generate permutation lists for the different color channel reordering every frame. The communication module allows the video stream server and client to exchange seeds, and transmit the encrypted video frame data.

**Figure 5:**
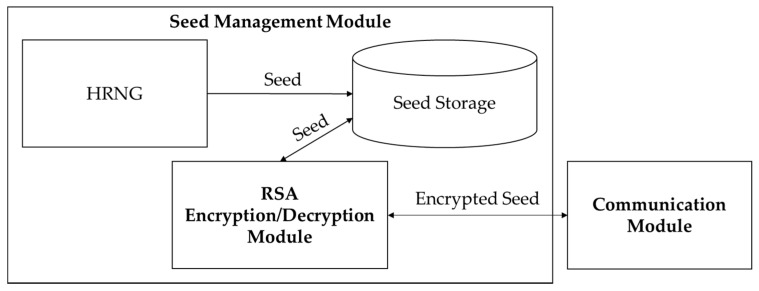
Seed management module structure and flows.

The seed management module includes a hardware random number generator (HRNG), seed storage, and RSA encryption/decryption module (see Figure 5). The seed management module uses HRNG to generate completely random 128-bit integer seed values. The generated seed value is stored in the seed storage. The seed storage must be managed to restrict external access because an attacker can replicate the entire permutation list by using it. RSA encryption/decryption module has different roles depending on the device to which the seed management module belongs. In the streaming server that generates seeds, and encrypts and transmits the video, the RSA encryption/decryption module encrypts the seeds using the public key of the client, and forwards them to the communications module. In the client that receives encrypted seeds from the server and decrypts the video, the RSA encryption/decryption module decrypts the received encrypted seeds using its private key and stores the decrypted seed values in the seed store.

**Figure 6:**
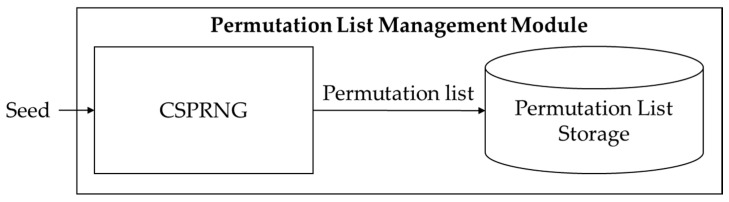
Permutation list management module structure and flows.

**Figure 7:**
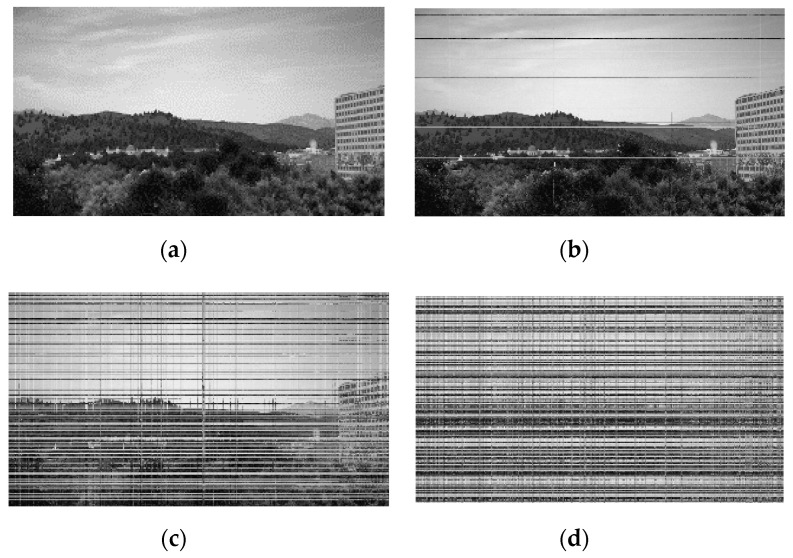
Original video frame and encrypted video frames: (**a**) original video frame and (**b**) 15 times permutated video frame. A human can easily recognize the frame content; (**c**) 126 times permutated video frame and (**d**) 307 times permutated video frame. A human cannot recognize the frame content. It has an MSE value of 100 when compared to the original frame.

The permutation list management module includes CSPRNG and permutation list storage (see Figure 6). CSPRNG generates a permutation list based on seed values generated by the seed management module. Most modern operating systems such as Microsoft Windows or Linux include the embedded CSPRNG module [22]. However, if the video stream server and client use a different operating system, the CSPRNG of each device may not be compatible. In this instance, the proposed algorithm can use cross-platform supporting external CSPRNG libraries such as GnuTLS (i.e., GNU transport layer security library) [23]. The permutation list is stored in the permutation list storage. The resolution of the input video determines the length of the permutation list. When the number of permutations is 15, the original video frame and permutated video frame’s MSE is 60 (see Figure 7). MSE is 80 for 126 permutations, and 100 for 307 permutations, respectively. Experimentally, when the difference from the original frame is more than MSE 100, it is difficult for a human to recognize the original contents of the video frame. In this instance, the length of the permutation list is similar to the number of horizontal pixels in the video frame. When the matrix permutation for one video frame is completed in the image processing module, a random number generated by CSPRNG is added to the permutation list to create a new list for the next frame. By updating the permutation list for each frame, the proposed algorithm can be robust against known-plaintext attacks.

CSPRNG updates the permutation list (see Algorithm 1). The minimum and maximum updated values are determined by the row and column length of the video frame. If the updated elements of the permutation list are negative, the maximum value is added. If they exceed the maximum value, the maximum value is subtracted. This brings the new values within the matrix size range of the video frame. CSPRNG is set to follow a continuous uniform distribution. This ensures that the elements of the permutation list are not concentrated in a specific range. The permutation list following the continuous uniform distribution can encrypt all frames to a similar level since the distribution characteristic is maintained after the permutation list is updated. By having the CSPRNG update the permutation list, updating the permutation list for every frame without using additional memory becomes possible.

The image processing module loads and performs matrix permutation on the video frame using the permutation list. Before matrix permutation, the color channels are separated, and matrix permutation for each channel is performed. The permutated channels are then merged to produce the final cryptographic video frame. Owing to the separated color channels and a different matrix permutation on each channel, an attacker cannot verify the color composition of the original video frames. The communication module exchanges the seed and encrypted video stream with other devices.
**Algorithm 1:** Permutation List Update.Input: Frame width *w,* height *h,* and permutation list *P*Output: Updated permutation list P01: for *p* in *P* do02:    if index of *p* % 2 == 0, then03:     *r* = random(*-w, w*)04:    else, then05:     *r* = random(*-h, h*)06:    end if07:    *p* = *p* + *r*08:    if index of *p* % 2 == 0, then09:     if *p > w* then10:      *p = p – w*11:     else if *p < 0*, then12:      *p = p + w*13:     end if14:    else, then15:     if *p > h*, then16:      *p – h*17:     elseif *p < 0*, *then*18:      *p + h*19:     end if20:    end if21: end for

### 3.2. Encryption Flow

**Figure 8:**
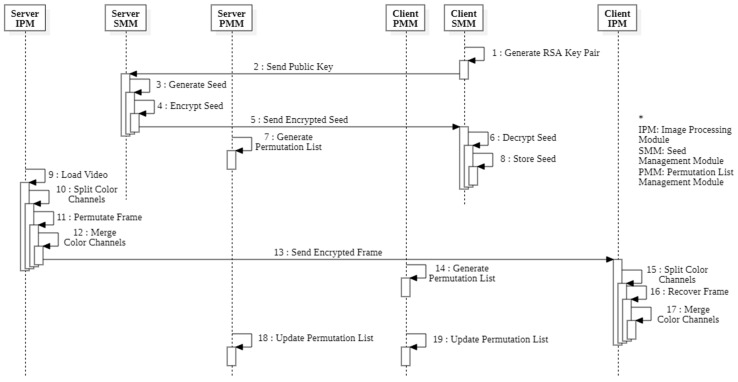
Proposed algorithm encryption sequence diagram.

The encryption and streaming process can be divided into five phases: connection establishment, video stream encryption, transmission, video stream decryption, and permutation list update (see Figure 8). The server encrypts and transmits encrypted videos, and the client receives encrypted videos from the server. In the connection establishment phase, (1) the client creates an RSA key pair and (2) transmits the public key to the video server. The video server that receives the streaming request (3) generates a random seed, (4) encrypts the seed with the public key sent by the client, and (5) sends the encrypted seed back to the client. (6) The client decrypts the transmitted seed and (7) stores it in the seed management module to establish the connection. Through processes (1)–(6), the server and client share the same seed. Seed exchanges using RSA infrastructure lead to relatively higher overheads. However, seed exchanges are performed only once during the initial connection. Thus, RSA-based seed exchange enables safe seed exchange without affecting actual encryption performance. In the video stream encryption phase, (8) the server loads the video from a file or video capture device and (9) generates a permutation list using the seed. Then, (10) the color channels are separated and (11) matrix permutation is performed for each channel. When the matrix permutation for all color channels is completed, (12) the channels are merged to produce the final encrypted video. At this step, the video frame separated by RGB can be merged in the order of BGR, GRB, etc. to make the color composition of the encrypted video frame different from the original video frame. (13) Encrypted frames are sent to the client. In the video decryption phase, (14) the client generates the same permutation list as the server using the seed. Then, (15) the color channels are separated and (16) matrix permutation is performed in the reverse order of the permutation list. (17) Finally, the original video frame is obtained by merging the color channels. At this time, the order of channel combinations should be the same as the server. After the original video frame is obtained, both the server and the client perform a permutation list update. (18) The permutation list is updated by adding a random number generated by CSPRNG to it. (19) The client updates the permutation list in the same way.

The proposed algorithm is designed to be robust to known plain-text attacks by applying different permutation lists to all frames. If the encryption module creates a new permutation list for each frame, the permutation list updating process may result in storage and processing overheads. The proposed algorithm is designed to increase storage space and speed efficiency, and to be robust against known-plaintext attacks by updating the permutation list using CSPRNG instead of pre-generating the corresponding permutation list for each frame.

## 4. Performance Evaluation

For the JLVEA encryption module’s performance evaluation, we first implemented it and then performed a security analysis, encryption speed analysis, and memory efficiency analysis. The proposed algorithm along with Liu and Koenig’s [14] and Sultana and Shubhangi’s [15] algorithms were implemented in Python, and RSA encryption was performed using PyCrypto [24], the cryptographic library for Python. All experiments were performed on the BCM2837 processor [25] included in Raspberry Pi (see Table 3). The experiment used a 1280 × 720 resolution video taken with the Raspberry Pi camera module.

In order to analyze the security of the proposed algorithm, the video frame encrypted with the algorithm of [14,15] and the video frame encrypted with the proposed algorithm were compared; when using the proposed algorithm, the contents of the original video frame were encrypted to a higher level with a higher MSE value. To show the efficiency of the proposed algorithm, we compared the encryption time. In order to show the memory efficiency of the permutation list update using CSPRNG, we generated a permutation list for a certain number of frames and compared the memory usage. The transmission failure rate was analyzed according to the communication loss rate to show that a pre-compression encryption method can more effectively preserve the original video frame following a communication loss than a post-compression encryption.

### 4.1. Security Evaluation

**Figure 9:**
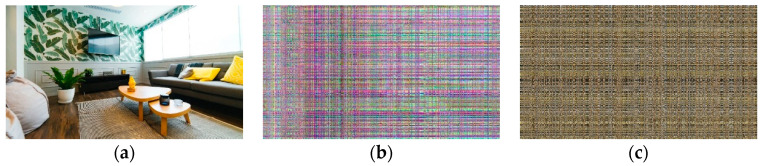
Color composition comparison for the proposed algorithm, and Sultana and Shubhangi’s algorithm [15]. Both frames are encrypted by performing 1920 permutations for rows and columns: (**a**) original video frame. The resolution is 1920 × 1080; (**b**) video frame encrypted with JLVEA. The frame has a different color composition than the original frame since the proposed algorithm separates color channels before permutation; (**c**) video frame encrypted with faro perfect algorithm. The frame’s color composition is similar to the original frame.

**Figure 10:**
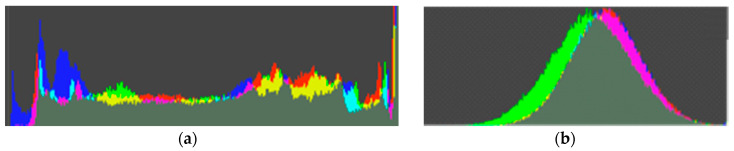
Color histogram comparison of original frame and frame encrypted with the proposed algorithm: (**a**) color histogram of original frame. It has its own specific color distribution; (**b**) color histogram of encrypted frame. Original color distribution has been destroyed.

The original frame has its own color composition according to the information contained within the frame. In the case of encrypted frames, the original color composition was completely removed by color channel separation and recombination. If the encrypted frame has a color composition similar to the original frame, the attackers may deduce some information in the original video frame, such as whether someone resides at their home or not. This experimental result shows the difficulty of information deduction based on color composition on the proposed algorithm. (See Figure 9 and Figure 10)

The permutation list also has a critical impact on security. The proposed algorithm can generate 47,483,761,585,029,120,000 different permutation lists. It takes 55 days to generate all permutation lists with a 10 TFlops computing machine. Moreover, since the frames encrypted by the proposed algorithm did not include internal file structures such as header markers or EOFs, it was not possible to automate the verification that they have been normally decrypted. It means all 47,483,761,585,029,120,000 frames reassembled through a brute-force attack must be verified directly by humans. Therefore, it was practically impossible to decrypt frames encrypted with the proposed algorithm through a permutation list brute-force attack. The attacker could deduce the permutation list if the attacker would succeed to extort an encrypted frame and the corresponding original frame. However, even if the extortion happens in the proposed algorithm, the attacker cannot decrypt the next frame because it does not use the same permutation list used for the previous frame. Liu and Koenig’s algorithm [14] can generate 256! permutation lists. However, this encrypted data contains internal file structures, which can be used to recover parts of the original data. Sultana and Shubhangi’s algorithm [15] uses only two types of faro shuffle and three types of frame rotation to generate a permutation list. Thus, only 2×3×1920=11,520 different permutation lists can be generated for 1920 permutations.

The seed should be strongly protected to ensure the security described above. Seed exchange was conducted only once for the whole encryption process. Thus, we adopted a public key infrastructure-based key exchanging mechanism to exchange the seed without considering the encryption overhead. We could use security proven algorithms such as RSA [26] or ECDSA [27].

### 4.2. Encryption Time Analysis

**Figure 11:**
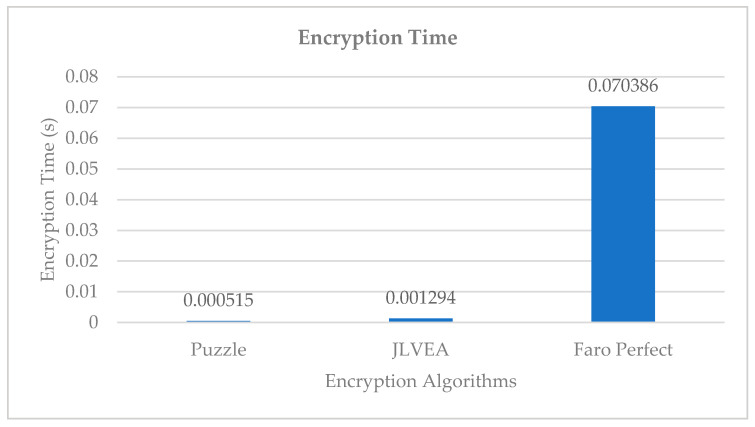
Encryption time.

We compared the encryption time of the proposed algorithm, Liu and Koenig’s algorithm [14], and Sultana and Shubhangi’s algorithm [15] with the same number of permutations (see Figure 11). Encryption with Liu and Koenig’s algorithm took 0.000515 s, the proposed algorithm took 0.001294 s, and Sultana and Shubhangi’s algorithm took 0.070386 s. Since Liu and Koenig’s algorithm involves post-compression encryption, the original video data to be encrypted is relatively small. Therefore, this algorithm can encrypt the video relatively faster. Sultana and Shubhangi’s algorithm, which performs pre-compression encryption in a manner similar to the proposed algorithm, showed a higher encryption time than the proposed algorithm. For Sultana and Shubhangi’s algorithm, the encryption time is assumed to be high because all rows and columns in a video frame must be rearranged for every permutation. Although the proposed algorithm takes longer to encrypt than Liu and Koenig’s algorithm, the level of overhead increase is acceptable because it is robust to header inference attacks and frame loss from packet loss. Moreover, its encryption performance is significantly better than the existing pre-compression encryption algorithms.

### 4.3. Memory Efficiency Analysis

**Figure 12:**
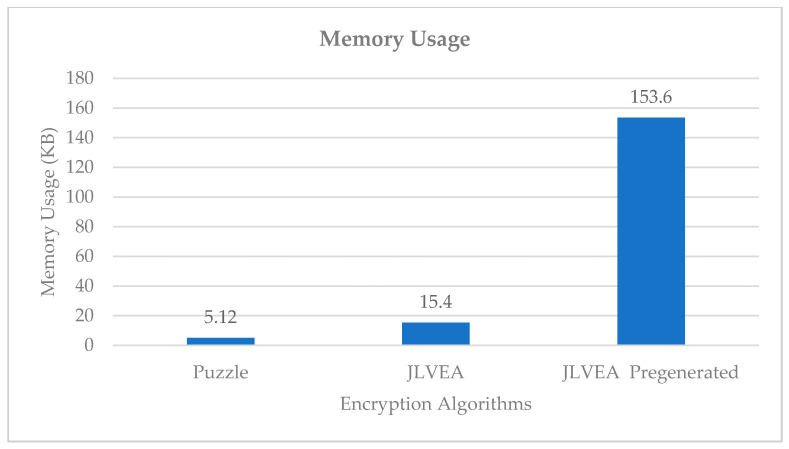
Memory usage.

We compared the memory usage of Liu and Koenig’s algorithm [14], the proposed algorithm, and the proposed algorithm with a pre-generated permutation list (see Figure 12). Liu and Koenig’s algorithm used 5.12 KB of memory, while the proposed algorithm used 15.4 KB of memory. The memory usage of the proposed algorithm with a pre-generated permutation list was 153.5 KB. The higher memory usage of the proposed algorithm than Liu and Koenig’s algorithm could be attributed to an increase in the number of the permutation lists because of color channel separation. However, since color channel separation resulted in a meaningful level of security improvement, the memory usage increase at that level was significant. The proposed algorithm could prevent an extreme increase in memory usage by using CSPRNG to update the permutation list instead of generating multiple permutation lists for each frame.

### 4.4. Communication Loss Resistance

**Figure 13:**
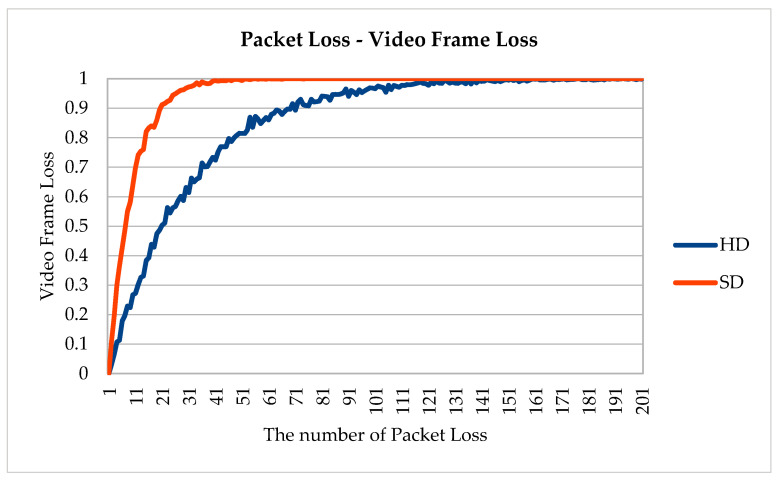
Packet loss–video frame loss.

**Figure 14:**
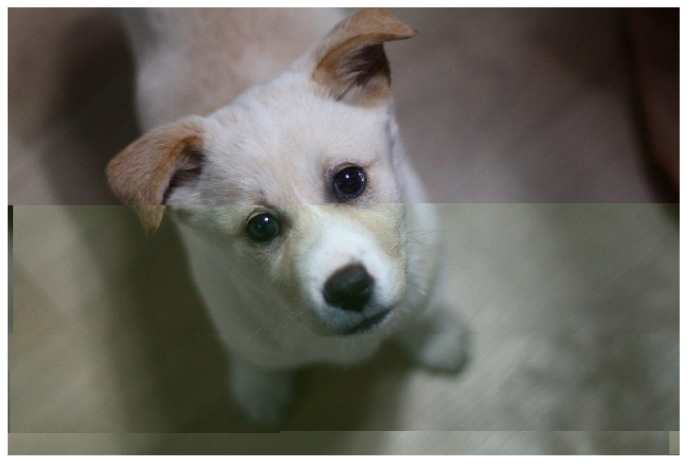
Video frame obtained by client following packet loss.

We simulated a packet loss scenario to check how many frames became unrecognizable in post-compression encryption algorithms and pre-compression encryption algorithms (see Figure 13 and Figure 14). Liu and Koenig’s algorithm [14] involves post-compression video encryption, which includes an internal file structure in the permutation range. Thus, if a packet containing an internal file structure is lost, the entire video frame cannot be recognized. For HD videos with a resolution of 1280 × 720, the original video frame could not be recognized with a high probability of 95% or more with 90 packet losses, and 50% of the original frame could not be recognized with just 22 packet losses. For standard definition (SD) videos with 640 × 480 resolution, because of a higher proportion of an internal file structure, the original video frame could not be recognized with a 95% probability for 28 packet losses and a 50% probability for 9 packet losses.

In the proposed algorithm, a part of the video frame was misplaced or lost because of packet loss. However, the contents of the video frame can be fully recognized. These experimental results show that post-compression encryption algorithm for real-time video streaming was vulnerable to packet loss. On the other hand, the proposed algorithm could recognize the content of video frame except the lost parts even when some packets are lost.

## 5. Conclusions

In this paper, we proposed a lightweight permutation-based video encryption algorithm to encrypt and transmit a video steam in real time on low-performance devices, such as IoT security cameras. Based on the permutation-based video encryption mechanism, we proposed an improved algorithm for a higher encryption performance than block cipher, while also being robust against problems such as known-plaintext attacks, header inference, and packet loss vulnerability from previously proposed permutation-based video encryption algorithms. Using CSPRNG, we solved the known-plaintext attack vulnerability by creating a new permutation list for each frame, and achieved a high security level through color channel separation, making it more difficult to deduce the contents of the video frame. Moreover, a permutation-based encryption was applied before compression so that the content of the original video frame could be recognized except for the lost part, even with packet loss during real-time video streaming. In the future, we would like to conduct further studies with the aim of minimizing the increased encryption time to the level of post-compression encryption algorithms.

## Figures and Tables

**Table 1:** Permutation-based video encryption algorithms.

	Liu and Koenig [14]	Sultana and Shubhangi [15]	Akhter et al. [20]
Encryption Position	Post-compression	Pre-compression	While-compression
Weakness	File internal information-based Inference, Streaming Overhead	Permutation list brute-force, Known-plaintext attack	Can adapt to MPEG only, Known-plaintext attack
Key Exchange	Each frame	Once	Once
Video Format Generality	Yes	Yes	No

**Table 2:** Example of color channel reordering information.

SEED Mod 5	Color Channel Merging Order
0	RBG
1	BGR
2	BRG
3	GRB
4	GBR

**Table 3:** Experiment environment.

Hardware Component	Specification
CPU	ARMv8 64 bit 1.4 GHz
SoC	BCM2837
RAM	1 GB LPDDR2
Networking	Gigabit Ethernet, 802.11 b/g/n/ac WLAN
